# Crosstalk between T lymphocyte and extracellular matrix in tumor microenvironment

**DOI:** 10.3389/fimmu.2024.1340702

**Published:** 2024-04-16

**Authors:** Die Lv, Yujie Fei, Hongli Chen, Junfeng Wang, Wenwen Han, Bomiao Cui, Yun Feng, Ping Zhang, Jiao Chen

**Affiliations:** State Key Laboratory of Oral Diseases and National Center for Stomatology and National Clinical Research Center for Oral Diseases, West China Hospital of Stomatology, Sichuan University, Chengdu, Sichuan, China

**Keywords:** extracellular matrix, tumor microenvironment, T lymphocytes, immune escape, targeted therapy

## Abstract

The extracellular matrix (ECM) is a complex three-dimensional structure composed of proteins, glycans, and proteoglycans, constituting a critical component of the tumor microenvironment. Complex interactions among immune cells, extracellular matrix, and tumor cells promote tumor development and metastasis, consequently influencing therapeutic efficacy. Hence, elucidating these interaction mechanisms is pivotal for precision cancer therapy. T lymphocytes are an important component of the immune system, exerting direct anti-tumor effects by attacking tumor cells or releasing lymphokines to enhance immune effects. The ECM significantly influences T cells function and infiltration within the tumor microenvironment, thereby impacting the behavior and biological characteristics of tumor cells. T cells are involved in regulating the synthesis, degradation, and remodeling of the extracellular matrix through the secretion of cytokines and enzymes. As a result, it affects the proliferation and invasive ability of tumor cells as well as the efficacy of immunotherapy. This review discusses the mechanisms underlying T lymphocyte-ECM interactions in the tumor immune microenvironment and their potential application in immunotherapy. It provides novel insights for the development of innovative tumor therapeutic strategies and drug.

## 1 Introduction

The tumor microenvironment (TME) is a complex ecosystem including tumor cells, immune cells, mesenchymal stromal cells, and ECM (1). It has become a hot research topic in recent years because of its key role in immunomodulation (2), angiogenesis (3), metabolic regulation, and ECM remodeling (4, 5). ECM is an important part of the tumor microenvironment and serves as a supportive architecture around the cells, allowing them to attach, migrate, and interact with each other. ECM molecules in the tumor microenvironment can affect cell proliferation, differentiation, migration, and immune escape, and play an important role in tumor cell immunotherapy.

Studies have shown that remodeling of tumor ECM will regulate the immune system, and tumor-associated ECM helps form an immunosuppressive network that acts as a barrier to protect cancer from treatment and promotes the malignant progression of tumors (6). In the complex tumor microenvironment, the continuous degradation and remodeling of ECM have important effects on the proliferation, migration, and signal transduction activities of T cells (7, 8). The ECM can also inhibit the antitumor effects of T cells by secreting inhibitory factors or regulating the function of immune cells (9).

At the same time, T cells can regulate ECM by releasing a variety of proteases and cytokines, which play a key role in anti-tumor immunotherapy (10, 11). T cells activate collagen synthesis and secretion of fibroblasts by secreting cytokines such as interferon-gamma (IFN-γ) and tumor necrosis factor-α (TNF-α), promoting collagen deposition and increased matrix stiffness (12, 13). T cells can also produce fibroblast growth factor (FGF) and transforming growth factor β (TGF-β), which induce fibroblasts to secrete collagen and hyaluronic acid, thereby altering the structure and composition of the ECM (14). In this paper, the interaction mechanism between ECM and T cells in the tumor microenvironment and its impact on tumor therapy was comprehensively described, which will help to deeply understand the complexity of the tumor microenvironment and provide new ideas and targets for future tumor treatment strategies.

## 2 ECM in the tumor microenvironment

**Figure 1:**
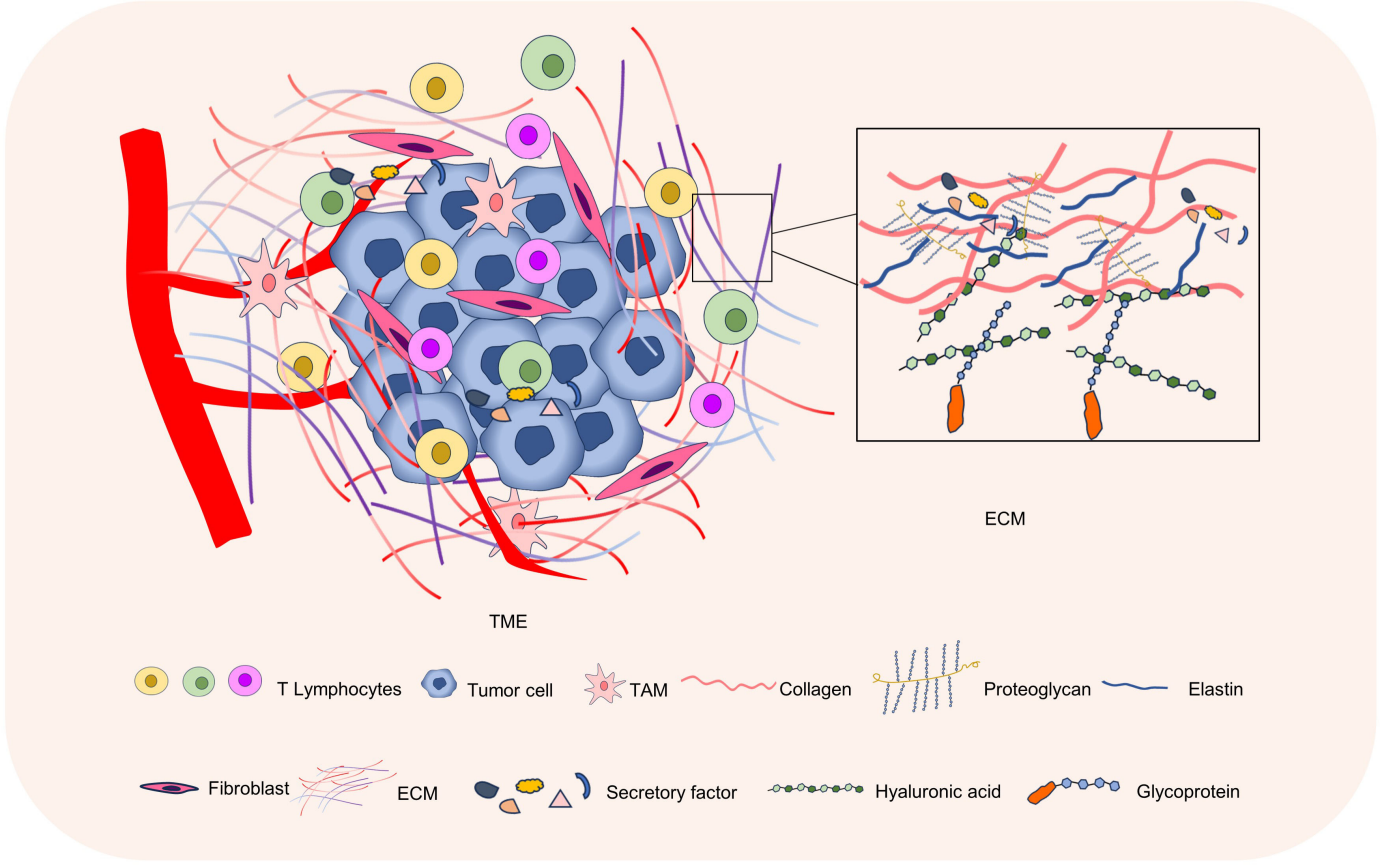
The ECM in the tumor microenvironment. The tumor microenvironment mainly consists of tumor cells, immune cells, stromal cells, and ECM. The ECM is composed of proteins, glycans, and proteoglycans, and is an important part of the tumor microenvironment.

The ECM plays an important regulatory role in the tumor microenvironment (15). The composition, structure, and physical properties of the ECM, as well as its interactions with tumor cells and other cells, have an impact on tumor growth, invasiveness, and metastatic ability (16, 17). The special microenvironmental state of the tumor, such as hypoxia, PH, etc., also affects the degradation and remodeling of ECM. Further study of the ECM in the tumor microenvironment can help to better understand the biology of tumors and provide new targets and strategies for the development of tumor therapies (**Figure 1**).

### 2.1 ECM components

The ECM is mainly composed of protein, proteoglycan, and glycosaminoglycan (18). Proteins mainly include collagen, elastin, fibronectin (FN), laminin, and proteoglycan, among which collagen is the most abundant protein in the ECM, forming fibers and providing strength and toughness to tissues (19). In the tumor microenvironment, collagen deposition increases gradually due to the inflammatory response and the active secretion of tumor cells (20). Abnormal collagen deposition can create an immunosuppressive environment that prevents the infiltration and activity of immune cells, thus weakening the ability to attack tumor cells (21). Glycosaminoglycan (GAG) is a kind of macromolecular polysaccharide, such as hyaluronic acid, chondroitin sulfate, etc (22). Studies have shown that tumor cells can produce more GAGs, and their structure and composition may also be different from normal tissues, and these changes may lead to an increase in tumor growth, metastasis, invasion, and immune escape (23, 24). In addition, the ECM also contains a variety of secreted proteins, including cytokines, chemokines, and growth factors, which may be involved in immune cell regulation (25). The type, content, and arrangement of these the ECM components can influence the behavior of tumor cells and tumor progression. The ECM can be divided into basement membrane and interstitial matrix (26). The mesenchymal matrix, which is mainly secreted by cells, is a loose network structure of collagen fibers, mainly composed of type I and type III collagen, fibronectin, elastin, and various proteoglycans (26). The basement membrane is a dense sheet protein network structure composed of type IV collagen, laminin, nestin, and heparan sulfate proteoglycan, which separates cells from the surrounding matrix and acts as a barrier for material transport (26, 27).

### 2.2 ECM structure and stiffness

Tumor development can lead to changes in ECM structure and stiffness (26, 28). The ECM is a complex structure that is dynamically remodeled through the synthesis and degradation of ECM proteins (9). Firstly, tumor cells secrete enzymes, such as metalloproteinases and glycoenzymes, that degrade ECM and change its stiffness triggers mechanotransduction signals to stimulate matrix metalloproteinase (MMP) secretion by cancer and stromal cells, elevated MMP activity promotes ECM component degradation and reorganization (29). Secondly, the proliferation and spread of tumor cells will cause the ECM to be tightly packed and increased, making the ECM more rigid (30). ECM with increased stiffness can activate signaling pathways in tumor cells, promote cell proliferation, migration, and invasion, and thus promote tumor growth and spread (31, 32). At the same time, the structural changes of ECM can limit the invasion and migration of T cells and prevent the infiltration of immune cells into the tumor, thus weakening the immune response to the tumor. Studies have found that the increased rigidity of ECM will lead to impaired function of immune cells, such as the activation and proliferation ability of T cells, and the antigen-presenting ability of dendritic cells. This can cause immune cells to be less effective in fighting tumors. Therefore, developing methods to modulate the structure and stiffness of ECM to improve the infiltration and activity of immune cells is critical to improving the effectiveness of tumor immunotherapy.

### 2.3 Interaction between cells within the TME and the ECM

The interaction between stromal cells, immune cells, tumor cells, and the extracellular matrix (ECM) forms a robust tumor barrier, significantly impacting therapeutic efficacy against cancer (1, 33). Stromal cells can synthesize and secrete important components of the ECM such as collagen, fibronectin, and are one of the main sources of ECM (34, 35). They participate in the formation and regulation of the tumor microenvironment by regulating the degradation and remodeling processes of the ECM, influencing tumor growth, invasion, and metastasis (36, 37). Cancer-associated fibroblasts (CAFs) play a significant role in the formation, progression, and invasion of tumors and can also impact the function of immune cells (38, 39). When discussing the degradation and remodeling of the ECM, matrix metalloproteinases (MMPs) and lysyl oxidase (LOX) have to be mentioned, as they play important roles in the tumor microenvironment (40–42). During tumor development, MMPs promote tumor cell migration by degrading collagen and fibronectin, allowing them to penetrate the matrix barriers and invade surrounding tissues and blood vessels (43). CAFs degrade and remodel the ECM by producing MMPs and activating FAK to limit the infiltration of effector immune cells while increasing the recruitment of inhibitory immune cells (such as Tregs, myeloid-derived suppressor cells (MDSCs), and TAMs), thereby suppressing the initiation of immune responses (44). Immune cells in the tumor microenvironment also play an important role in the degradation and remodeling of ECM (45), with tumor-associated macrophages (TAMs) being the most common immune inhibitory cells, secreting factors that suppress immune responses, inhibit T cell activity, and promote tumor growth and metastasis (46). LOX primarily functions in collagen cross-linking, thereby increasing the stability and mechanical strength of the extracellular matrix (47). In the tumor microenvironment, LOX expression is typically significantly increased, leading to abnormal matrix stiffening and fibrosis (48). CAFs derived from oral squamous cell carcinoma (OSCC) release sEVs containing a large amount of active LOX, which preferentially bind to ECM inducing collagen cross-linking, thereby promoting epithelial-mesenchymal transition (EMT) (49). Additionally, increased secretion of LOX inhibits CD8+ T cells infiltration, thereby reducing the effectiveness of immunotherapy (50). Immunotherapy attempts to attack tumor cells by activating the patient’s immune system. However, the presence of ECM and the immunosuppressive effects of stromal cells can prevent immune cell infiltration into the tumor area, limiting the effectiveness of immunotherapy (51). Therefore, targeting the interaction between ECM and TME cells in the tumor microenvironment is an important research direction for cancer treatment, including targeting MMPs and LOX to disrupt the fibrous network surrounding the tumor and promote immune cells infiltration (52, 53). Inhibiting the immunosuppressive effects of stromal cells, such as by targeting TAMs or CAFs to reduce their numbers or alter their function, and combining tumor microenvironment modulation with immunotherapy are combined treatment strategies to improve therapeutic efficacy (54).

### 2.4 ECM receptors in T cells

**Figure 2:**
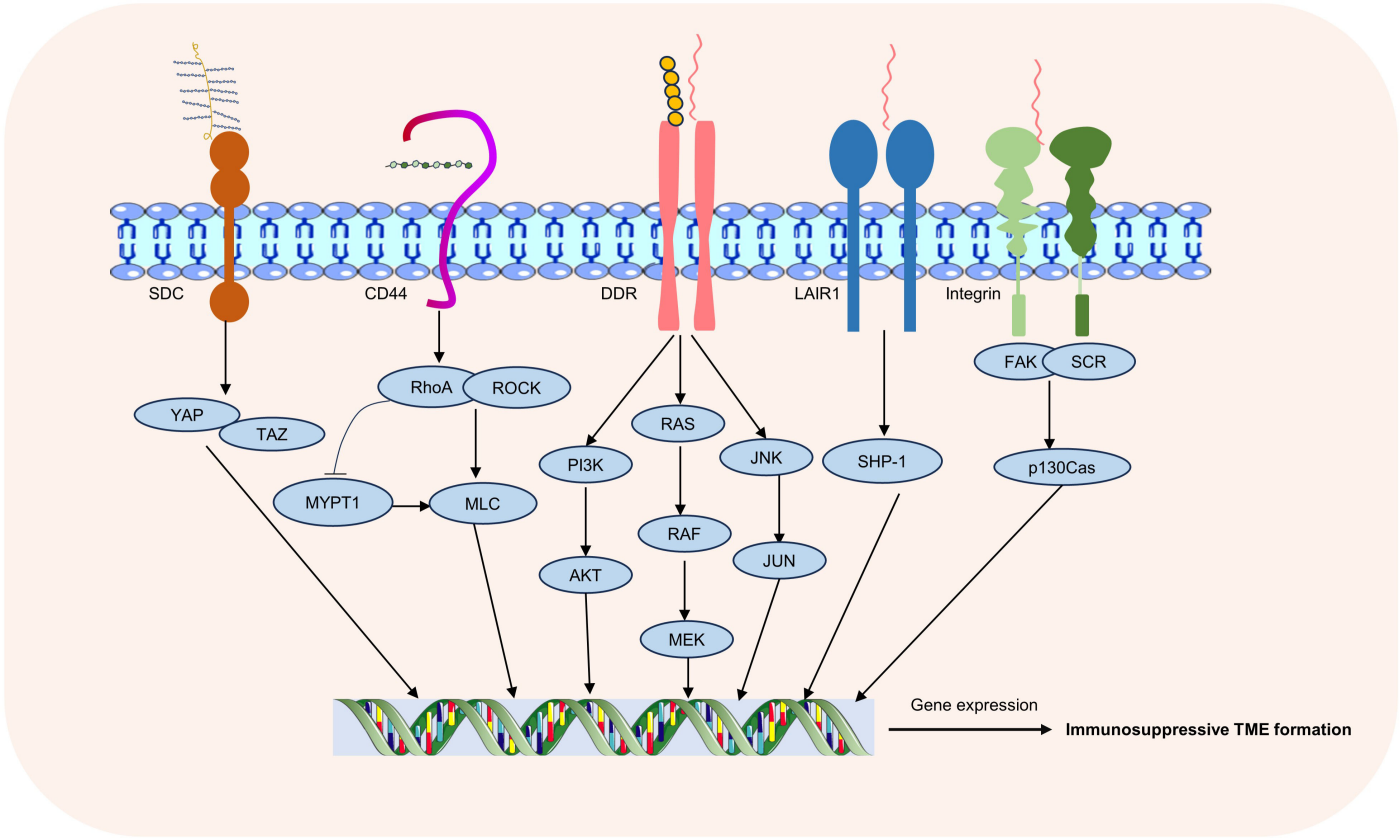
ECM receptors and associated signaling pathways. Common receptors in T-cell-ECM interactions include integrins, DDR, LAIR1, CD44, and SDC, through which changes in the extracellular matrix regulate multiple intracellular signaling pathways.

T cells express a variety of ECM receptors, including integrin, discoidin domain receptor (DDR), leukocyte-associated immunoglobulin-like receptor 1 (LAIR1), CD44, syndecan (SDC) (7, 55–57). These receptors bind to ECM components and regulate T cell migration, activation, and function (9). Integrins such as LFA-1 (CD11a/CD18) bind to intercellular adhesion molecules (ICAMs), promote the stability of T cells and antigen-presenting cells (APCs), contribute to immune synapse formation and T cells activation. VLA-4 (CD49d/CD29) can bind to vascular cell-adhesion molecule-1 (VCAM-1) and fibronectin, which are involved in T cells migration and tissue localization (58, 59). DDR, including DDR1 and DDR2 subtypes, regulates the physiological process of cells mainly by binding with collagen, and is involved in collagen deposition, ECM remodeling and tissue regeneration and repair (7). CD44 is a hyaluronic acid receptor that plays a role in T cell migration, activation, and polarization, and is critical for maintaining microenvironment localization and function of T cells (56). Collagen receptor LAIR1 is a transmembrane receptor of the immunoglobulin superfamily. Collagen deposition inhibits NK cells by inhibiting LAIR1 signaling and promotes tumor colonisation (60). In melanoma, LAIR1 deletion promotes its metastatic growth, LAIR1 expression is associated with improved clinical outcomes in human metastatic melanomas, and LAIR-1 may be a promising cancer therapeutic target (61). SDC is a class of transmembrane glycoproteins, including four members: SDC1, SDC2, SDC3 and SDC4, and its ligands include proteoglycans, fibronectin, cytokines and growth factors. The interactions of these ligands with syndecan are critical for biological processes such as ECM formation, cell adhesion, and cell signaling (57) (**Figure 2**).

## 3 Interaction between ECM and T lymphocytes in tumors microenvironment

### 3.1 Effect of the ECM on T lymphocyte activation and proliferation

Undesirable alterations in the ECM within cancer contribute to the development of a profoundly immunosuppressive tumor microenvironment, subsequently impacting the proliferation and activation of T cells (62). Under physiological conditions, the extracellular matrix (ECM) serves as a scaffold and conduit for immune cells. However, within the tumor microenvironment (TME), ECM composition and structure frequently undergo alterations, potentially diminishing T cell activation and impeding migration. Consequently, this hampers T cell-mediated tumor cytotoxicity. RNA sequencing (RNAseq) analysis revealed that Col4 suppressed the transcription of activated T cell genes while enhancing the expression of immunosuppressive cytokines, thereby diminishing T cell efficacy against tumors (63). Intestinal cancer organoids and CAFs co-culture can spontaneously organize into superstructures with high shrinkage and sclerotic ECM ability, and this co-culture mode significantly inhibits the proliferation of T cells, leading to the generation of immunosuppressor microenvironments (64). On the other hand, the absence of the ECM components may also lead to malignant progression of tumors, col1 deletion in myofibroblasts is associated with increased number of MDSCs, which specifically expresses high levels of CD206, F4/80, arginase-1, CCL2, and interleukin-18. These MDSCs further suppress the function of T and B lymphocytes through arginase-1 and CD206, potentially contributing to the immunosuppressive microenvironment in pancreatic ductal adenocarcinoma (PDAC) (65). In addition, the ECM can directly or indirectly regulate T cell proliferation by interacting with T cell receptor signaling pathways (66). In breast cancer, the collagen receptor DDR1 inhibits T cells activation and infiltration by promoting collagen fiber alignment and further contributes to tumor immune escape (6). The ECM also provides interactions between T cells and other cell types and influences the activation status of T cells through intercellular signaling pathways (67). The ECM impacts the activation status and proliferation rate of T cells through various mechanisms, thereby influencing the tumor immune response (68). Understanding how the ECM modulates T cells function in the tumor microenvironment can offer crucial insights for the development of cancer treatment strategies.

### 3.2 Effect of the ECM on T lymphocyte infiltration and migration

The efficient migration of effector T cells to the tumor site is closely associated with the prognosis and efficacy of cancer immunotherapy. However, due to abnormal alterations in the ECM within the tumor microenvironment, the migration and infiltration of T cells are frequently restricted (8, 69). The ECM provides support for cell adhesion and regulates cell movement through its structure and stiffness (70). Under physiological conditions, the structure and stiffness of the ECM provide appropriate signals to T cells, thereby maintaining their normal migration. However, in certain cancers, alterations occur in the structure and stiffness of the ECM, leading to the formation of an abnormal tissue known as tumor mesenchyme. This aberrant ECM may restrict T cells infiltration and migration, consequently dampening the immune response (71). Collagen develops resistance to programmed cell death protein 1/programmed cell death ligand 1 (PD-1/PD-L1) immunotherapy in lung tumors through up-regulation of LAI R1 expression and downstream signaling, and reduction of tumor collagen deposition through lysyl oxidase like 2 (LOXL2) inhibition increases T cell infiltration, reduces depletion of T cells, and eliminates resistance against PD-1/PD-L1 (72). Interfering with collagen stability reduces ECM content and tumor hardness, thereby improving T cells migration and enhancing the efficacy of anti-PD-1 blocking (8). In the tumor microenvironment, besides cancer cells, numerous other cell types are present, including fibroblasts and immune cells. These cells have the capacity to modify the properties of the ECM and regulate T cell migration and infiltration either through cytokine secretion or direct interaction with T cells (73). In hepatocellular carcinoma (HCC), SPP1-positive macrophages and CAFs participate in the formation of the ECM, promoting the formation of TIB (Tumor immune barrier) structures, and preventing CD8^+^ T cells from infiltrating into TIB-coated tumors (74). In addition, MMPs, as a key enzyme regulating ECM, also plays an important role in T cell infiltration. The high expression of MMP1 has been confirmed in various types of cancers, indicating poorer overall survival rates, and showing significant negative correlations with the quantities of CD8+ T cells, CD4+ T cells, and macrophage infiltration, suggesting MMP1 as a potential novel biomarker for immunotherapy (75, 76). Furthermore, MMP1 expression correlates with dendritic cell (DC) markers HLA-DQB1, HLA-DRA, and HLA-DPA. DCs can increase tumor metastasis levels by enhancing Treg responses and inhibiting the cytotoxicity of CD8+ T cells, consistent with high levels of Treg markers FOXP3, CCR8, STAT5B, and TGFβ (75). In melanoma, MMP2 promotes CAFs infiltration to regulate the ECM, thereby suppressing CD8+ T cells infiltration. Targeting MMP2 can improve the tumor microenvironment and enhance sensitivity to immunotherapy (77). Most studies indicate a positive correlation between MMP2 and Treg infiltration, serving as a typical cancer immunotherapy inhibitor (78). Interestingly, research has found that Tregs inhibit the production of MMP2 in the early stages of tumor development to control the invasion and migration processes, possibly related to the dual nature of Tregs (79). Therefore, ECM plays an important role in regulating T cells migration and infiltration.

### 3.3 Effect of the ECM on T lymphocyte function

Throughout cancer development, alterations in the structural and physical characteristics of the ECM can disrupt the normal function of T cells, potentially leading to detrimental effects on immune system responses (80). Differential expression of surface markers on T cells reflects differences in T cell function. The CD8 molecule typically serves as a marker for cytotoxic T cells (CTLs), which play important roles in immune surveillance and clearing abnormal cells by recognizing and eliminating infected pathogens or cancer cells through the CD8 receptor on their surface (81). The collagen density in the tumor microenvironment may reduce the cytotoxic activity of tumor-infiltrating immune cells, suppress the tumor-killing function of CTLs, and support tumor cell immune escape by regulating CD8+ T cell nuclear size and the expression of related genes (82, 83). The CD4 molecule is commonly used to identify helper T cells (Th cells), with Th1 cells primarily activating macrophages and cytotoxic T cells by producing interferon-gamma, while Th2 cells promote the proliferation and differentiation of B cells, leading to antibody production. Additionally, Th17 cells primarily produce inflammatory factors such as IL-17, participating in the regulation of inflammatory responses (84). In the tumor microenvironment, the levels of Th1 and Th17 cells are negatively correlated with collagen content, while the levels of Th2 and Treg cells are positively correlated with collagen content, with lower collagen content possibly associated with longer disease-free survival (85). The FoxP3 marks regulatory T cells, which possess suppressive functions that can modulate immune responses, maintain immune balance, and prevent autoimmune reactions (86). ECM can influence the number and distribution of Tregs in tumor tissues, with increased levels of ECM-related proteins potentially promoting the recruitment and aggregation of Tregs, affecting their differentiation and activity, thereby inhibiting immune cell activity and promoting tumor immune escape (87, 88). Immune checkpoint molecules such as PD-1 and cytotoxic T-lymphocyte antigen 4 (CTLA-4) are typically used to inhibit T cell activity, and the ECM in the tumor microenvironment may enhance the expression of PD-1 on T cell surfaces by interacting with cell surface receptors such as integrins and CD44, thereby promoting tumor immune escape (89, 90). The latest research shows that by changing the viscoelasticity of its surrounding the ECM, it is possible to produce functionally different T cell populations from T cells that receive the same stimulus (91). In addition, proteases, chemokines, and cytokines in ECM can directly interfere with the physiological function of T cells and are considered to be one of the main factors of tumor immune tolerance. By binding to T cell surface receptors, these factors inhibit the proliferation, differentiation, and production of effector molecules of T cells, thus weakening the ability of T cells to kill tumor cells (72). In conclusion, alterations in the ECM significantly impact T cell function during tumor development. Hence, comprehending the mechanisms underlying ECM-T cell interactions could aid in the development of novel tumor immunotherapy strategies.

### 3.4 Regulation of the ECM by T cells

**Figure 3:**
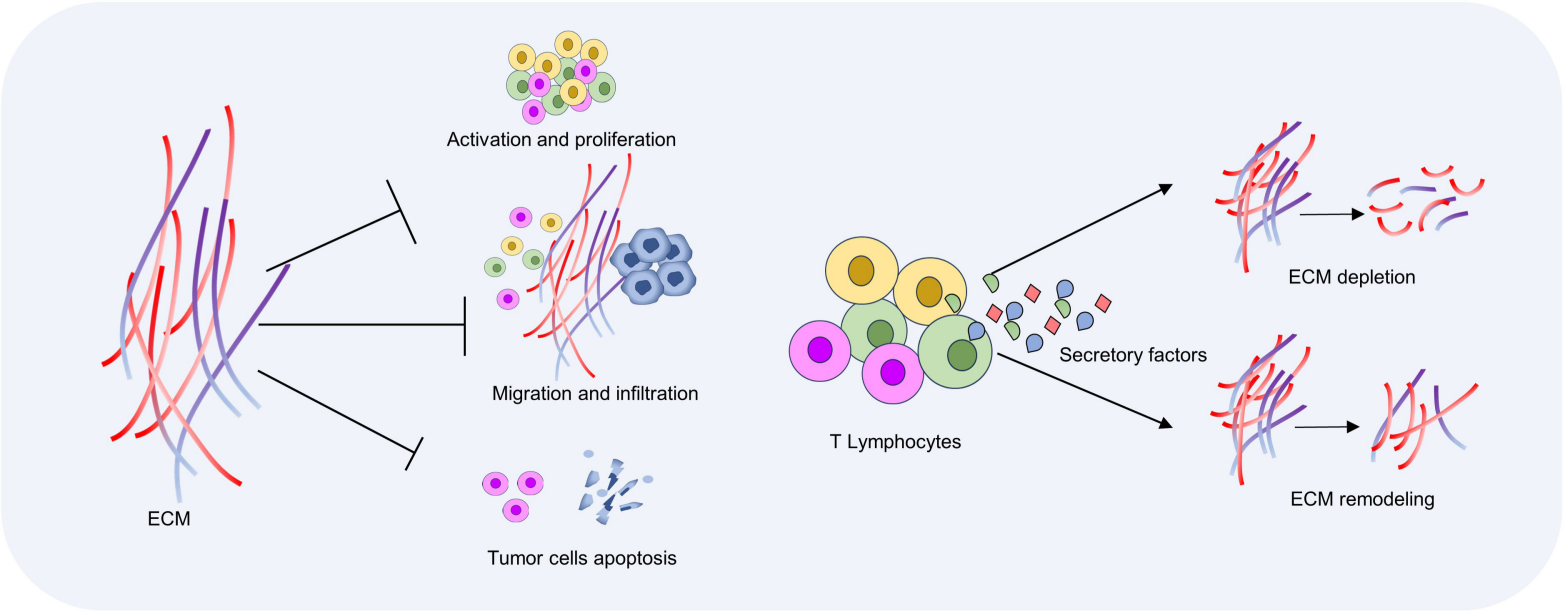
Interactions between T cells and ECM. ECM density and hardness increase in the tumor microenvironment, which inhibits T cell activation and migration. T cells induce ECM degradation and remodeling by secreting chemokines and cytokines, and then regulate anti-tumor immune response.

In cancer, activated T cells within the tumor microenvironment can secrete a variety of proteases, including metalloproteinases and lysosomal enzymes. These proteases degrade major ECM components such as collagen and fibrin, thereby altering the physical properties of the tumor microenvironment (92). MMP affects tissue integrity, immune cell recruitment, and tissue renewal by degrading ECM components and releasing matrix components, cell surface-bound cytokines, growth factors, or their receptors (41). The ECM itself provides the signals needed for cell growth, migration, and survival, and is involved in the process of tumor development and metastasis (93). By degrading ECM, T cells can disrupt the link between tumor cells and ECM, interfere with tumor cell signaling, and thus inhibit cancer progression (94). On the other hand, immune cells secrete chemokines and cytokines CXC, IFN-γ, TNF-α, IL4, and IL13 to activate collagen synthesis and secretion of fibroblasts, thereby promoting collagen deposition and matrix stiffness. IL-13 is believed to affect the synthesis and degradation of ECM, and thus participate in the process of tissue repair and fibrosis. T cells can directly or indirectly affect the function of fibroblasts by secreting IL-13, and promote the production of more ECM protein in fibroblasts. In addition, T cells can also regulate the production and arrangement of collagen when they come into contact with fibroblasts. These factors can promote the synthesis and deposition of ECM, thereby increasing the adhesion of cancer cells to the surrounding environment and enhancing the ability of cancer cells to infiltrate and metastasize (95, 96) (**Figure 3**).

## 4 Emerging therapeutic strategies targeting the ECM

**Figure 4:**
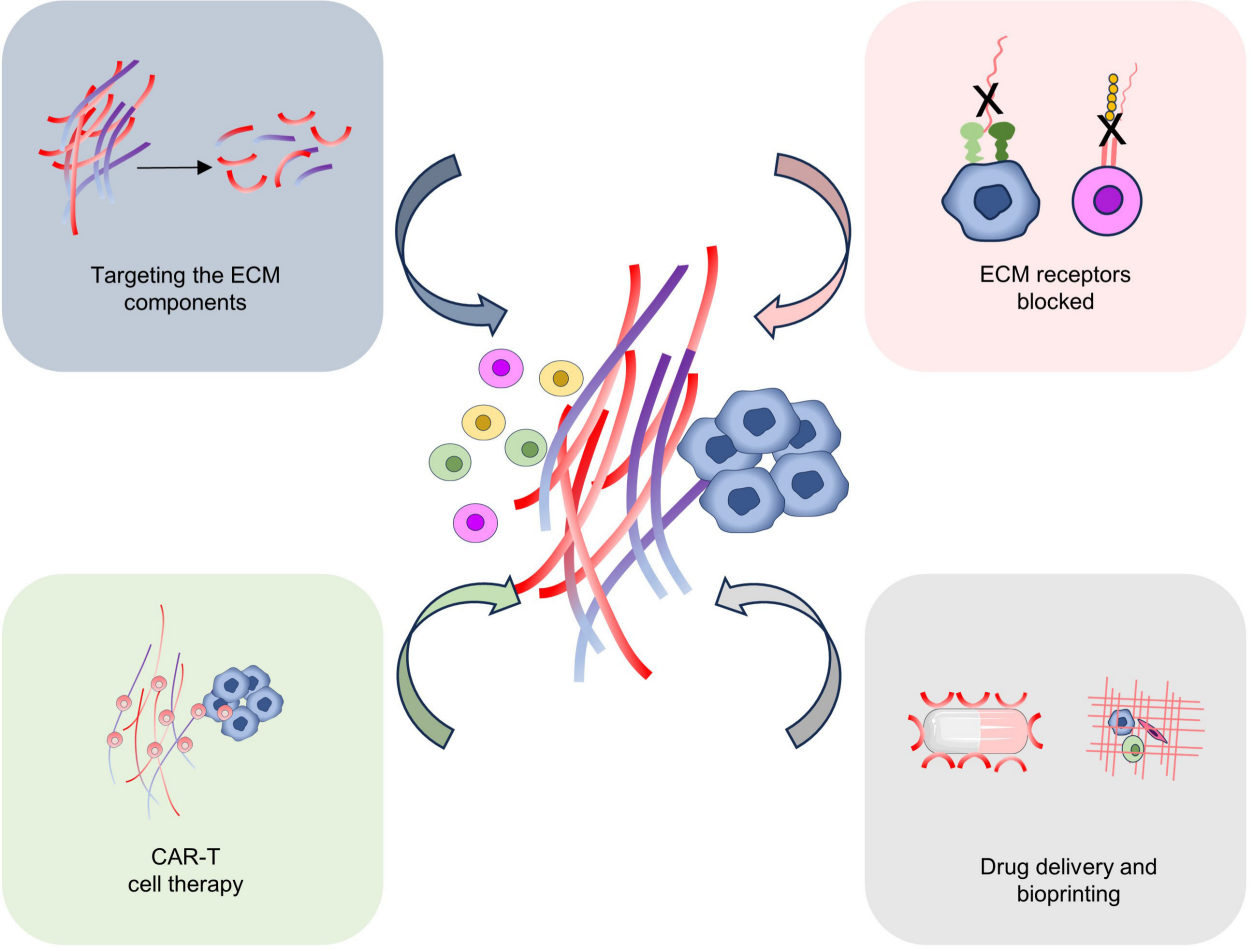
Targeting ECM for tumor therapy. Several possible strategies for targeting ECM for tumor treatment include targeting the ECM components, ECM-related receptor blocked, CAR-T cell therapy, drug delivery, and Bioprinting.

Research into targeting the ECM for tumor treatment is a rapidly expanding field. Throughout tumor development, the ECM can serve as a protective barrier against immune attack, diminish immune cell infiltration, and facilitate tumor growth and metastasis (20). By utilizing specific antibodies or other biomolecules to disrupt the interaction between the ECM and tumor cells (15). For example, antibodies are used to block proteins in the ECM, such as fibronectin or hyaluronic acid, thereby interfering with adhesion and signaling between tumor cells and the ECM (97). These approaches are anticipated to decrease the invasion and migration of tumor cells while enhancing the capacity of immune cells to infiltrate tumors, thereby inhibiting tumor growth and metastasis (6). In addition, several studies have focused on developing ECM-related drug delivery systems to improve the efficacy of anti-tumor drugs. By binding drugs to molecules that interact with ECMs, the enrichment of drugs in tumor tissues can be increased and their release time can be prolonged. This strategy is expected to improve the local efficacy of the drug while reducing toxicity to healthy tissue (98). The ECM itself can serve as a promising anti-tumor therapeutic target. By modifying the composition or structure of the ECM, it is possible to modulate the tumor microenvironment and influence the growth and metastatic potential of tumor cells. Therefore, targeting the ECM has the potential to alter the composition and structure of the ECM within the tumor microenvironment, augment the infiltration and cytotoxicity of immune cells, and enhance the efficacy of tumor immunotherapy (9, 12) (**Figure 4**).

### 4.1 Targeting the ECM components

More and more evidence suggests that the ECM, a non-cellular component of the tumor microenvironment, plays a significant role in modulating responses to immunotherapy (99–101). Collagen is the most abundant ECM protein in the tumor microenvironment across various solid tumor types and plays a crucial role in tumor progression and metastasis. Recent studies indicate that collagen directly contributes to resistance to immune checkpoint inhibitors (ICIs), therefore, targeting collagen may be a potential strategy to improve the efficacy of tumor immunotherapy (72, 102) (103–105). Strategies targeting the ECM, particularly collagen proteins, have been proposed in combination with immune checkpoint inhibitors, showing enhanced efficacy at least in murine models (106). LOX is a key inducer of ECM rigidity, inhibition of LOX enzyme activity, combined with anti-PD-1 administration, improved the infiltration and accumulation of effector CD8+ T cells within tumors and increased the efficacy of PD-1 blockade in a PDAC mouse model (8). Loss of the highly expressed MRC2 collagen receptor within the immunosuppressive the ECM myCAF subtype promoted CD8+ T cell infiltration and enhanced sensitivity to immune checkpoint inhibitor (ICI) in a murine model of breast tumors (107).

Hiroaki Wakimoto et al. detected the oncolytic adenovirus ICOVIR17, which expresses hyaluronidase, mediating both degradation of HA within the ECM and subsequent alteration of the immune landscape of the TME. This study provides mechanistic insights into combined immunotherapy with PD-L1/PD-1 blockade, reshaping both innate and adaptive immune cells (108). PDAC is considered a highly immune-suppressive and heterogeneous tumor (109, 110). Immunotherapy with immune checkpoint inhibitors (targeting CTLA4, PD1, PDL1) has not been very successful in treating PDAC (109, 110). The hexosamine biosynthetic pathway (HBP) is a diverting pathway of glycolysis (111, 112). Serving as a metabolic node in cancer cells that, on the one hand, promotes survival pathways and, on the other, affects the synthesis of hyaluronic acid in ECM (113–115). Sharma et al. found that a small molecule glutamine analog (6-diazo-5-oxo-L-norleucine [DON]) reduced the self-renewal potential and metastatic ability of tumor cells (116). Moreover, the DON treatment reduced hyaluronic acid and collagen in the tumor microenvironment, leading to extensive the ECM remodeling and increased infiltration of CD8+ T cells. Additionally, DON treatment rendered pancreatic tumors sensitive to anti-PD1 therapy, resulting in tumor regression and extended survival (116).

### 4.2 Target the ECM receptors and ECM-related cytokine

Targeting ECM-related receptors on the cell membrane and cytokines that regulate ECM contribute to enhancing anti-tumor immune response. Integrin and DDR are both important ECM receptors and promising and challenging targets for the treatment of a variety of diseases (117, 118). Current research findings indicate that mechanical forces originating from the ECM regulate the stemness and cell cycle of breast cancer cells via the integrin and DDR signaling pathways, thereby promoting tumor proliferation. Targeting both integrin and DDR has been shown to yield enhanced therapeutic effects on tumors (119). The co-inactivation of DDR1 and integrin can effectively reduce the radio resistance of human glioblastoma cells (120). In addition, simultaneous targeting of EGFR and integrin αvβ3 receptors can effectively regulate tumor cells and the tumor ECM. This approach reduces F-actin levels in tumor cells, decreases collagen content within the tumor ECM, and enhances drug permeability (121). Therefore, ECM receptor multi-target combination therapy may produce better efficacy. CD44, a multifunctional receptor involved in cell-cell and cell-ECM interactions, is expressed at high levels in many cancer cell types and their metastases. Some tumors, such as glioblastomas, express only standard CD44, while others, including gastrointestinal cancers, bladder cancer, cervical cancer, breast cancer, and non-Hodgkin lymphoma, also express CD44 variants (122). Shalom et al. found that injecting reagents that disrupt CD44-ligand interactions (such as CD44s- or CD44v-specific antibodies) inhibited local tumor growth and metastatic spread in animal models (122).

Tumor cells and their tumor microenvironment (TME) cells secrete various factors, with proteoglycans further activating these factors, which may be crucial for interactions between tumor cells and their TME (123). Most proteoglycan can act as cell factor receptors, playing a significant role in the localization of cytokines (124, 125). Heparan sulfate proteoglycans (HSPGs) act as reservoirs for cytokines by binding to them, thereby controlling the availability and mobility of these biomolecules. On cell surfaces, HSPGs act as co-receptors, mediating interactions between cytokines and their receptors by binding to ligands and receptors (126). A key function of HSPGs is to regulate the expression and function of cytokines, chemokines, growth factors, morphogens, and adhesion molecules. They can act as ligands or co-receptors for various signaling transduction receptors, affecting pathways such as FGF, VEGF, chemokines, integrins, Wnt, Notch, IL-6/JAK-STAT3, and NF-κB, promoting tumor malignancy (127). Additionally, proteoglycan-stimulated human dendritic cells (DCs) produce highly suppressive regulatory T cells (Tregs) through mechanisms involving metabolic reprogramming, PD-L1, IL-10, and IDO (128). Collagen I is one of the main components of the ECM, with tumors in the tumor microenvironment containing more type I collagen compared to healthy tissues. The increase in these collagens can elevate the concentration of nearby cytokines, promoting Treg cell infiltration by upregulating the expression of CD4 and FOXP3, as well as the percentage of CD4+/FOXP3+ T cells, thereby inducing immune-suppressive TME (129–131). Thus, by regulating the synthesis and secretion of proteoglycans and collagen I, tumors can increase the concentration of nearby cytokines, influencing the immune response and tumor growth in the tumor microenvironment.

MMPs can impact immunotherapy by modulating the profile of cytokines residing in the ECM. In benign tumors, MMP expression levels are typically low and participate in physiological ECM turnover. However, in malignant tumors, MMP expression is dysregulated and often increased, leading to excessive degradation of ECM components and alterations in the cytokine profile. MMPs can modulate the function of certain growth factors and cytokines, promoting tumor growth (132). In immunotherapy, targeting MMPs may significantly enhance the cytotoxicity of T cells (CD8+ CTLs) and the ratio of cytotoxic T cells/regulatory T cells (CD8+ CTLs/Tregs) and cytokine secretion by inhibiting the production of pro-inflammatory cytokines and promoting the production of anti-inflammatory cytokines, synergistically eliminating primary tumor growth and effectively inhibiting tumor metastasis (133, 134). Yeow et al. engineered a TNFα recombinant protein fused with a CSG peptide ligand that binds to laminin-nidogen complexes (98, 135). The TNFα-CSG complex reduces tumor hardness and expands tumor vessels by activating immune cells to release ECM-degrading proteases, thereby enhancing tumor perfusion and augmenting the uptake of contrast agents (gadolinium and iron oxide nanoparticles) within the tumor (98). Horn and colleagues assessed the combined inhibition of TGF-β, PD-L1, and LAIR-1 signals in a cancer mouse tumor model. This strategy reduced collagen content in the ECM, enhanced infiltration of activated CD8+ T cells, and decreased tumor growth (70, 106). The composition and organization of tumor ECM impact drug delivery to tumor cells (136, 137). Furthermore, targeting TGF-β has proven effective in softening ECM as TGF-β signals ECM cell stiffening (98). Chen et al. attempted to promote cytotoxic T-cell infiltration and drug entry into the TME using multifunctional nanoparticles (98, 138). They engineered approximately 40-nanometer nanoparticles of heparin-cysteine 5.5/l-arginine (HFCA), which spontaneously release nitric oxide (NO) and load chemotherapeutic drug docetaxel (DTX) and immune checkpoint inhibitor anti-PD1, enhancing the effectiveness of drugs to kill cancer cells and evade the immunosuppressive TME. NO contributes to tumor vessel normalization (98, 139) and activates matrix metalloproteinases to degrade collagen components of tumor ECM (140).

### 4.3 Enhancement of CAR-T cell therapy

CAR-T therapy relies on the engineered transportation of T cells to tumor sites, where the ECM serves as a physical barrier for CAR-T cell infiltration and direct contact with cancer cells (141, 142). Engineering T cells indirectly to modulate the ECM mechanism, giving them the ability to regulate ECM, holds promise for tumor-infiltrating T cells and drug penetration within tumors (98, 141). Mechanically priming CAR-T cells to gain the capacity for self-infiltration into the dense and aligned ECM of solid tumors represents a promising cellular engineering approach (98). Thus, manipulation of mechanosensitive mechanisms existing in the cell membrane (e.g., piezoelectric ion channels, integrins) or body (e.g., actin filaments, microtubules) can serve as suitable targets for improving T cell inclusion and migration (98).

Engineering CAR-T cells to express the gene encoding heparanase (HPSE) has been shown to promote T cell infiltration, thus demonstrating antitumor efficacy against matrix-rich solid tumors (98, 143). Fibroblast activation protein (FAP), a membrane-bound protease, targeted by CAR-T cells, effectively enhances the killing of tumor cells in vitro and is considered a suitable target for CAR-T cell therapy against immune-suppressive TME components (106, 144). Preliminary data from a Phase I clinical trial targeting FAP using CAR T cells for treating mesothelioma suggest the feasibility of this approach (106, 145). Expression of urokinase plasminogen activator receptor-associated protein (uPARAP/Endo180/CD280/MRC2) delineates a subset of stromal remodeling CAFs (107, 146). aiming to disrupt its role in TME remodeling, positioning MRC2 as a putative stromal target for solid tumor CAR T therapy (106). Nattokinase (NKase), a thrombolytic agent, has been found by Zhang et al. to, when injected intratumorally, not only degrade the main component of ECM, fibronectin but also inhibit the fibrosis produced by CAFs, thereby reducing tumor stiffness, enhancing perfusion, and alleviating hypoxia (147). In a xenograft human breast MDB-MA-231 tumor model, pre-treatment with NKase has been shown to facilitate the infiltration of CAR-T cells into the tumor, thus favoring tumor suppression (147).

### 4.4 Drug delivery

Solid tumors are typically composed of heterogeneous cells and an ECM arranged with high stiffness. The rigid ECM restricts the recruitment and infiltration of immune cells into the tumor while amplifying MDSCs in the tumor microenvironment, thereby promoting suppressive immunity (98). Stiff ECM is frequently targeted through various strategies to enhance T cell infiltration (98). Hence, novel technologies for tumor-specific immune therapeutic drug delivery systems are required.

According to reports, many drug carriers have increased the efficacy of immunotherapeutic agents by enhancing the circulation time of T cell activators, ICP inhibitors, and cytokinesis inhibitors, as well as the targeting capability of immune cells (69, 148). Tissue protease S (CatS) is significantly expressed in TAMs, DCs, and MDSCs within the TME. Selective inhibition of CatS via engineered nanocarriers using specific small molecule inhibitors or siRNA targeting TAMs, DCs, and MDSCs showed targeted delivery and functional release of inhibitors, offering new opportunities for effective adjunctive therapies to promote anti-tumor immunity (149). Collagenase, elastase, and hyaluronidase, among other matrix-modulating enzymes, have been utilized to promote the degradation of the ECM components, aiming to reduce tumor stiffness (106, 150). The rapid advancements in nanomedicine have introduced novel avenues for cancer immunotherapy. Nanoparticles have exhibited advantages over conventional drug delivery systems (67). In diverse tumor experimental models, collagenase-functionalized nanoparticles have been demonstrated to facilitate ECM degradation, thereby enhancing the permeability and retention of anti-tumor drugs (106, 151–154). Pan et al. co-delivered collagenase and trastuzumab into HER2-positive BT474 tumor-bearing mice using a thermosensitive hydrogel, promoting the penetration of therapeutic antibodies into deeper tumor tissues (106, 155). Dong et al. described nanoparticles loaded with chemotherapeutic doxorubicin and a NO donor. NO-induced the activation of MMPs within the TME, degrading collagen, and facilitating the penetration of nanoparticles and their therapeutic payload in an orthotopic 4T1 breast cancer model (106, 156).

The ECM acts as a critical barrier to the tumor infiltration of CTLs, impairing T cell-dependent immunotherapy for HCC. Tumor-acid-induced CaP dissolution promoted the release of IL-12 and HAase responsible for ECM digestion, enhancing tumor infiltration and CTL proliferation. Furthermore, MMP-2 overexpression triggered the in situ release of αPD-L1 in the tumor, preventing tumor cells from evading CTL-mediated killing. This combinatorial strategy induced robust anti-tumor immunity, effectively suppressing mouse HCC growth (94). Triple-negative breast cancer (TNBC), a particularly aggressive subtype, shows relative resistance to programmed cell death-1 (α-PD1) therapy (157, 158). Zhao et al. applied doxorubicin hydrochloride liposomes (Dox-L) as nanochemotherapy ICD induction and used losartan as a matrix-depletion agent to enhance the efficacy of α-PD1 (losartan + Dox-L + α-PD1). Results indicated that losartan reduced the ECM, facilitating enhanced delivery of Dox-L and further dendritic cell (DC) maturation. Fibroblast activation protein (FAP), overexpressed on CAFs, is considered a universal tumor-targeting antigen (159–161). Zhen et al. utilized ferritin (a compact nanoparticle protein cage) as a photosensitizer carrier and linked FAP-specific single-chain variable fragments (scFv) to the surface of ferritin. They found that nano-PIT inhibited the secretion of C-X-C motif chemokine ligand 12 (CXCL12) and ECM deposition regulated by untreated CAFs, mediating T cell exclusion and preventing physical contact between T cells and cancer cells (161).

### 4.5 Bioprinting

Bioprinting technology can be used to fabricate three-dimensional tumor models with complex structures, simulating the interaction between lymphocytes and the ECM within actual tumors. Such models aid in studying the influence of the ECM on lymphocyte activity and infiltration capabilities. T cells play a crucial role in adaptive immune responses within the body, especially in combating intracellular pathogens and cancer. Research into T cell activation often utilizes two-dimensional (2D) culture systems, which do not replicate the interactions between naturally activated cells and the ECM that affect activation (162, 163). Mechanical characterizations suggest that hydrogels have pathophysiologically relevant stiffness, mimicking the structure of lymph node tissues (164, 165). Joseph et al. found that using a mouse T cell lymphoma line EL4 or primary mouse T cells in 3D bioprinting, and activating them with a combination of 10ng/mL phorbol myristate acetate (PMA) and 0.1μM ionomycin, resulted in a 1.3-fold increase in the ratio of active EL4 cells in soft substrates compared to those in hard substrates (164). Primary mouse T cells activated with PMA and ionomycin exhibited 1.35 times more active cells in soft substrates compared to those in hard substrates. This demonstrated variations in T cell responses within the 3D bioprinting scaffold, faithfully reproducing T cell activation and revealing the pathophysiological characteristics of T cells in infectious biology, autoimmune diseases, and cancer (164).

## 5 Conclusion and challenges

This review summarizes the interactions between ECM and T cells in the tumor microenvironment, as well as potential anti-tumor immunotherapy strategies targeting ECM. Tumor cells and stromal cells modulate the ECM through secretion of various proteases and production of ECM components, impacting T cells activation and migration, among other functions. Conversely, T cells influence the anti-tumor immune response by releasing cytokines and chemokines that remodel the ECM. Intervening with the ECM surrounding the tumor can disrupt the interaction between tumor cells and their environment, thereby inhibiting tumor cell growth, dissemination, and metastasis. However, the ECM also serves vital physiological functions in normal tissues, necessitating ECM-targeted therapies to be specific enough to avoid adverse effects on normal tissues. Tumor heterogeneity, characterized by diverse cell types and matrix components within tumors, mandates individualized therapeutic approaches tailored to specific tumor types. Overall, targeting the ECM for anti-tumor immunity holds promise as a significant strategy for future cancer therapy. Nevertheless, further research and clinical validation are imperative to address challenges and assess effects across various tumor types and individuals.

## Author contributions

JC: Writing – original draft, Writing – review & editing. DL: Visualization, Writing – original draft, Writing – review & editing, Investigation. YJF: Visualization, Writing – original draft. HC: Investigation, Resources, Writing – review & editing. JW: Investigation, Writing – review & editing. WH: Investigation, Writing – review & editing. BC: Writing – review & editing. YF: Writing – review & editing. PZ: Writing – review & editing.
